# Osteoporotic Distal Fibula Fractures in the Elderly: How To Fix Them

**DOI:** 10.7759/cureus.6552

**Published:** 2020-01-03

**Authors:** Arnab Sain, Sitender Garg, Vijay Sharma, Umesh K Meena, Hemant Bansal

**Affiliations:** 1 Orthopaedics, All India Institute of Medical Sciences, New Delhi, IND

**Keywords:** osteoporotic, distal fibula, fracture, operative fixation

## Abstract

Osteoporotic fractures of the distal fibula in elderly patients is a challenge to manage. Non-operative management has a poor outcome so operative management is preferred. There are a variety of options for operative management such as locked plate systems, anti-glide plate construct, dual plating constructs, fibula nail, plate with tibial pro-fibular screws, and injectable bone cement (polymethylmethacrylate (PMMA), calcium phosphate). However, no clear guidelines exist for the operative management of osteoporotic distal fibula fractures. The surgeon should detect osteoporotic fractures early to make the best use of resources and avoid complications such as implant failure.

## Introduction and background

Osteoporosis is a systemic disease in which there is a deterioration of the microarchitecture of bone and the bone mass is low, which leads to the risk of fractures secondary to low-energy mechanisms. Fragility fractures of the ankle are increasing in incidence in the elderly population, especially among women [1-3]. The World Health Organization has defined osteoporosis as having a T-score of less than -2.5 (bone mineral density 2.5 SDs below the young adult average bone mineral density) obtained by dual-energy X-ray absorptiometry (DEXA) scan or by the presence of a fragility fracture [1]. Osteoporotic fractures are a challenge to treat due to the poor purchase of hardware. Fragility fractures of the weight-bearing lower extremities are difficult to manage [1].

Failure of fixation of the lateral side is more common than the medial side. The most common deformity seen in failed ankle fractures is lateral malleolus shortening and external rotation [4]. Non-operative management of ankle fractures has a high incidence of nonunion and malunion [5-7]. Better functional outcomes are seen with the operative treatment of ankle fractures in elderly patients [8-9].

This article will focus on operative management in osteoporotic distal fibula fractures, as they pose a greater challenge to manage than medial malleolus fractures.

## Review

Methods of operative fixation

Locking Plate System

A locking plate system is one of the most effective fixation techniques for osteoporotic fractures. The newer available plates have an increased number of options for locking screw placement in the distal fibula. These new versions of locking plates are pre-contoured, have a more anatomic fit, and are useful when there is significant comminution [1]. The locking plate required more torque to fail as compared to the conventional non-locking plate. Also, fixation with the locking plate was independent of bone mineral density [10]. The locking plates provide more rigid fixation with a more stable construct and are useful for multi-fragmentary fractures and patients with poor bone quality [11].

During the fixation of distal fibula fractures to avoid penetration of the ankle joint, screws in the distal fibula can obtain purchase in only a single cortex [1]. Kim et al. found that in cadaveric distal fibulas, locking plates required fewer unicortical screws than non-locking plates to achieve the same biomechanical stability [1,12].

**Figure 1:**
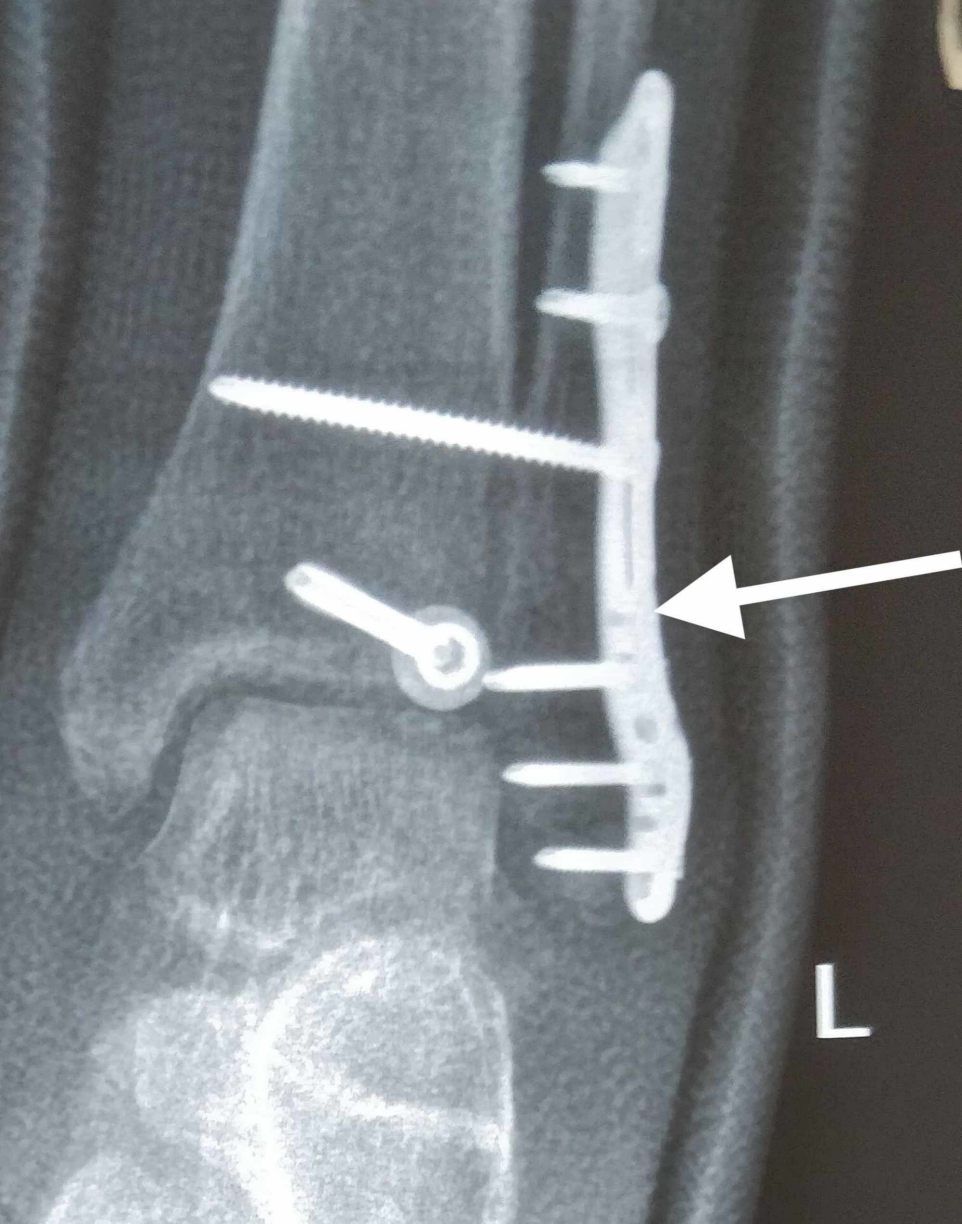
Locking plate system used in a patient with an osteoporotic distal fibula fracture (anteroposterior view)

**Figure 2:**
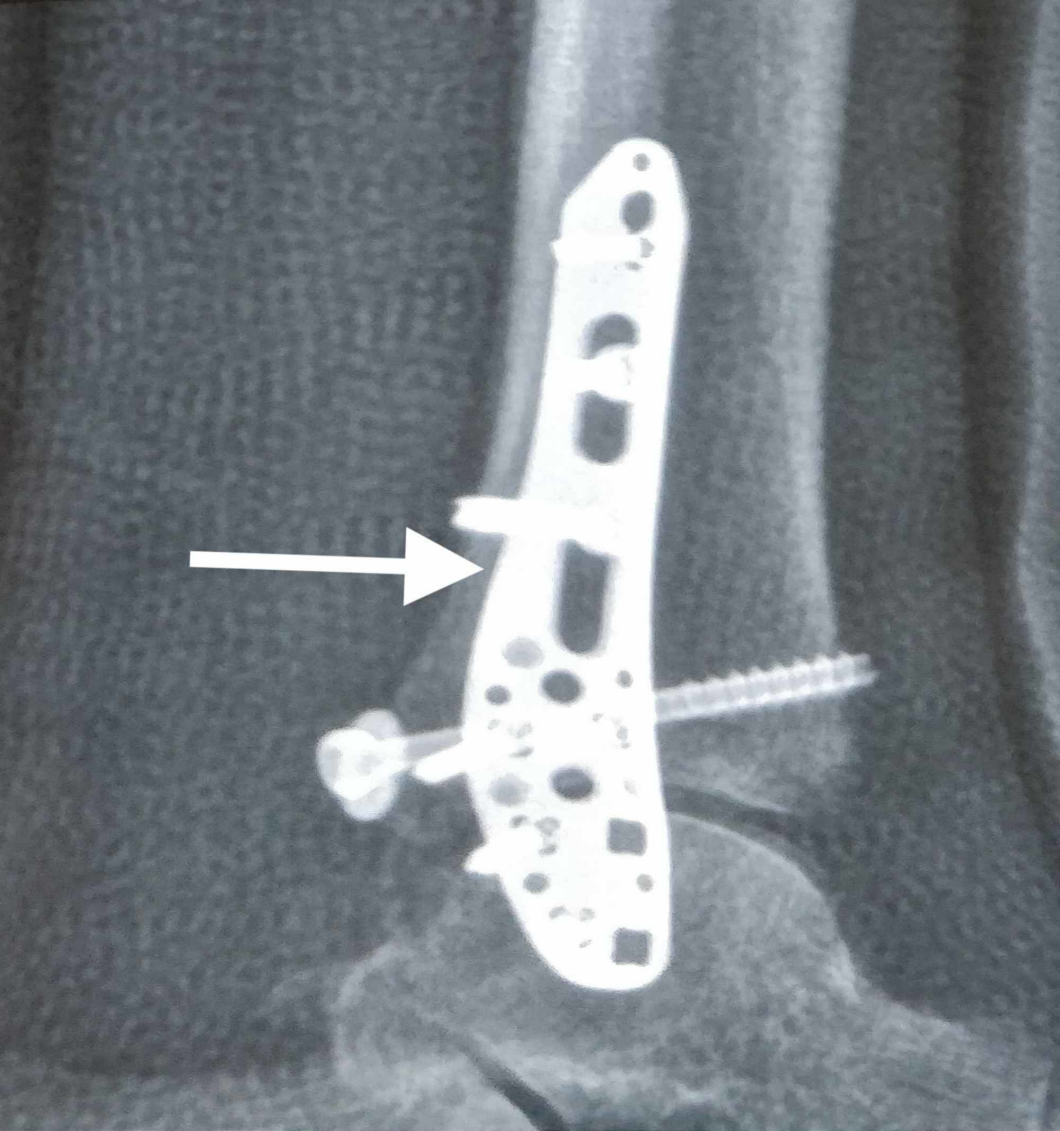
Locking plate system used in a patient with an osteoporotic distal fibula fracture (lateral view) Note that the locking plate has the option of putting screws in multiple planes distally, making it a stable construct.

The locking plate with improved biomechanical strength allows early mobility and fewer chances of implant failure [10,13]. The cost of a locking plate is higher than non-locking plates, but taking into account the chances of implant failure with non-locking systems, locking plates provide a more satisfactory option [11]. Below are images of the X-ray radiograph anteroposterior (AP) view and a lateral view showing the locking plate construct in osteoporotic distal fibula fracture (Figures 1-2).

Anti-glide/Posterior Plating

When the plate is placed on the posterior aspect of fibula, it is possible to have bicortical screws distally, but when the plate is placed on the lateral surface, only uni-cortical screws can be placed distally to avoid screw penetration of the joint. So posterior plate placement makes a more stable construct than lateral plate placement [1].

However, there is controversy in the comparison between the lateral locking plate system and the posterior anti-glide plate construct. According to Minihane et al., the anti-glide plating construct is best suited for an oblique fracture pattern and has greater strength as compared to a lateral locking plate system [1,14]. However, Switaj et al. demonstrated that a lateral locking plate is a better construct than a posterior anti-glide plate [15].

Schaffer et al. demonstrated that the posterior anti-glide plate has better biomechanical properties as compared to a lateral plate [16]. Also, in posterior plate placement, there is an opportunity for putting a compression screw across the fracture site in the oblique fracture pattern, making a more stable construct [1]. However, there is no difference in biomechanical properties between the poly-axial locking plate and the non-locking plate in anti-glide plate placement [17].

Lateral plating also leads to skin impingement by screw heads, leading to skin irritation and postoperative wound-healing problems [1]. However, posterior plate placement has a higher incidence of irritation of the peroneal tendon, leading to hardware removal for peroneal tendon lesions [18].

Dual Plating

**Figure 3:**
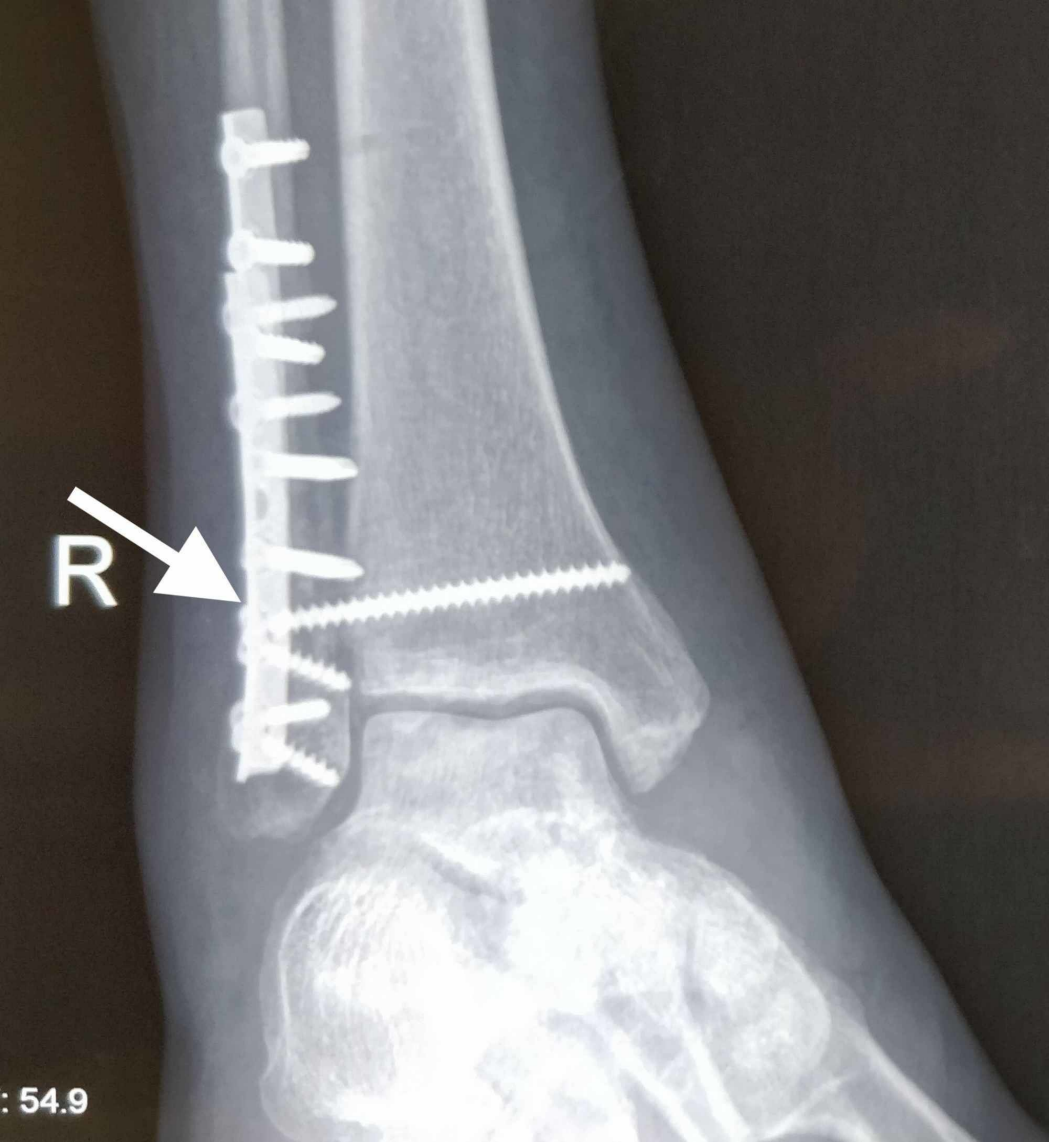
X-ray radiograph anteroposterior (AP) view showing the dual plating construct in an osteoporotic distal fibula fracture Note one plate applied on the lateral aspect of the fibula and the other on the posterior aspect of the fibula.

**Figure 4:**
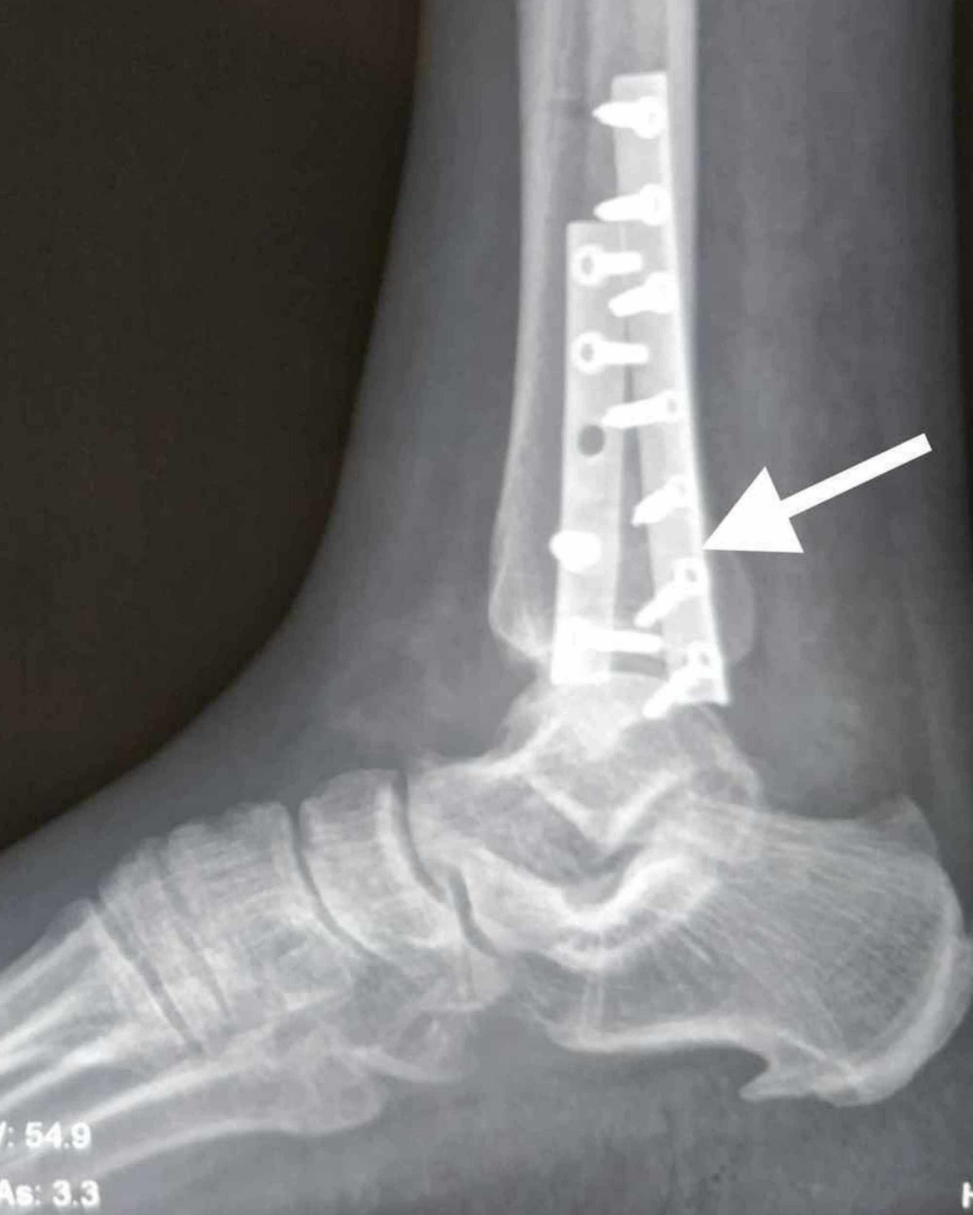
X-ray radiograph lateral view showing the dual plating construct in an osteoporotic distal fibula fracture Note one plate applied on the lateral aspect of the fibula and the other on the posterior aspect of the fibula.

In cases of osteoporotic fractures with significant comminution, the placement of two plates, one on the lateral aspect and the other on the posterior aspect of the fibula, provides a stable construct [1]. This method is advantageous because it allows for biplanar fixation with the use of non-locking plates. It is a cost-effective method of fixation in a comminuted distal fibula fracture [1,19]. Randall et al. found that dual plating is a relatively safe option with functional outcome comparable to the locking plate system, with a low incidence of implant failure [20]. Kwaadu et al. demonstrated that dual plating provides additional stability in complex fibular fractures due to advanced age or a higher energy injury and does not appear to increase the incidence of hardware removal due to skin or soft tissue irritation [21]. Also, in comminuted fractures, longer plates should be used to spread the stress load over a longer distance [1]. Below are the images of an X-ray radiograph in the anteroposterior (AP) and lateral views, showing the dual plating construct in an osteoporotic distal fibula fracture (Figures 3-4).

Fibula Nail/ Intramedullary Fixation

Osteoporotic bone has more chance of loss of purchase due to relatively weak bone leading to a less stable construct [1,22]. Also, using a larger diameter screw does not solve the problem [23]. To prevent this problem of screw pull-out, a fibular nail can be used. There are many advantages of using a fibular nail like a smaller incision, less soft tissue stripping, and less disruption of the fracture site biology, which promotes early healing. According to Rajeev et al., patients treated with a fibular nail had fewer complications and good restoration of function, leading to good patient satisfaction [1,24]. Bugler et al. used an Acumed fibular nail (Acumed, LLC, Hillsboro, Oregon) for unstable distal fibular fractures and found that it has good functional and radiological outcomes [25]. According to Jain et al., intramedullary fixation of unstable distal fibular fractures can give excellent results that are comparable with those of modern plating techniques [26]. Lee et al. compared the Knowles pin with the plate for the fixation of distal fibula fractures in the elderly and found that patients treated with a Knowles pin had less duration of hospital stay, less need for analgesic, and fewer complications such as hardware irritation as compared to internal fixation with a plate [27]. Appleton et al. suggested that a fibular nail provided a minimally invasive method of fixation of distal fibula fractures in the elderly, with fewer wound complications [28].

*Plate with Tibial Pro-fibular Screw*s

Panchbhavi et al. studied 16 patients with osteoporotic fractures treated with a hook plate with tibial pro-fibular screws and found excellent outcomes. This technique provided stable fixation for osteoporotic ankle fractures in elderly patients until the union was achieved with good functional outcomes [29].

In another study, Panchabhavi et al. investigated the use of tibia-pro-fibula screws and found that, compared with the same construct without the additional screws, the tibial pro-fibular screws resulted in a 9% increase in torque, a 24% increase in the amount of external rotation, and a 34% increase in energy before the construct failed. Thus, tibial pro-fibular screws provided a relatively easy, inexpensive method to increase the plate construct strength [30].

Injectable Bone Cement

Polymethylmethacrylate (PMMA) is useful in cases of severe osteoporosis and by increasing the density, it increases the pull-out strength of screws [1]. There are two methods of using PMMA cement.

*The first method involves removing the stripped screws from their holes, injecting the cement into the stripped screw holes, and reintroducing the screws into the holes but not completely tightening them. The cement is then allowed to set, and then, the screws are tightened [1,31].

*The second method involves introducing the cement inside the bone and allowing it to set completely before inserting the screw. The hardened cement can then be drilled and tapped before inserting the screw [1,31].

Although PMMA is inexpensive, it has poor biocompatibility and is also non-absorbable and causes thermal necrosis as it is exothermic. Also, it is difficult to remove during revision surgery [1,32].

An alternative to PMMA is calcium phosphate cement. It is more biocompatible and osteoconductive [1,32]. It is gradually replacing PMMA in traumatology [33,34]. Calcium phosphate increases the pull-out strength of screws in cancellous bone [35-36]. However, in a study by augmentation with tricalcium phosphate cement vs PMMA vs no augmentation, showed equal pull-out strengths (4 fold increase compared to no augmentation) between tricalcium phosphate cement and PMMA [37]. Panchbhavi et al. found that the use of calcium sulfate and calcium phosphate improved the pull-out strength of tibial pro-fibular screws in osteoporotic bone [38].

## Conclusions

For the treatment of osteoporotic distal fibula fractures, a variety of options are available but there is a lack of clear guidelines. The locking plate, posterior anti-glide plate, and fibula nail have better evidence of an advantage in osteoporotic bone. The use of dual plating and hook plate with tibial pro-fibular screw in osteoporotic fracture has less evidence. The use of injectable cement is found in some literature and calcium phosphate and other bio-absorbable cement are gradually replacing PMMA. Overall, the surgeon should diagnose osteoporotic fractures at the earliest and make the best use of available resources for the benefit of the patient and avoid complications like implant failure.
